# Tracking auditory mismatch negativity responses during full conscious state and coma

**DOI:** 10.3389/fneur.2023.1111691

**Published:** 2023-03-10

**Authors:** Adianes Herrera-Diaz, Rober Boshra, Paniz Tavakoli, Chia-Yu A. Lin, Netri Pajankar, Elham Bagheri, Richard Kolesar, Alison Fox-Robichaud, Cindy Hamielec, James P. Reilly, John F. Connolly

**Affiliations:** ^1^Centre for Advanced Research in Experimental and Applied Linguistics (ARiEAL), McMaster University, Hamilton, ON, Canada; ^2^Neuroscience Graduate Program, McMaster University, Hamilton, ON, Canada; ^3^Princenton Neuroscience Institute, Princeton University, Princeton, NJ, United States; ^4^School of Biomedical Engineering, McMaster University, Hamilton, ON, Canada; ^5^Department of Anesthesia, McMaster University, Hamilton, ON, Canada; ^6^Department of Medicine, McMaster University, Hamilton, ON, Canada; ^7^Critical Care Medicine, Hamilton Health Sciences, Hamilton, ON, Canada; ^8^Department of Psychology, Neuroscience & Behaviour, McMaster University, Hamilton, ON, Canada; ^9^VoxNeuro, Inc., Toronto, ON, Canada

**Keywords:** mismatch negativity, event-related potentials (ERP), coma, disorder of consciousness, brain injury

## Abstract

The mismatch negativity (MMN) is considered the electrophysiological change-detection response of the brain, and therefore a valuable clinical tool for monitoring functional changes associated with return to consciousness after severe brain injury. Using an auditory multi-deviant oddball paradigm, we tracked auditory MMN responses in seventeen healthy controls over a 12-h period, and in three comatose patients assessed over 24 h at two time points. We investigated whether the MMN responses show fluctuations in detectability over time in full conscious awareness, or whether such fluctuations are rather a feature of coma. Three methods of analysis were utilized to determine whether the MMN and subsequent event-related potential (ERP) components could be identified: traditional visual analysis, permutation *t*-test, and Bayesian analysis. The results showed that the MMN responses elicited to the duration deviant-stimuli are elicited and reliably detected over the course of several hours in healthy controls, at both group and single-subject levels. Preliminary findings in three comatose patients provide further evidence that the MMN is often present in coma, varying within a single patient from easily detectable to undetectable at different times. This highlights the fact that regular and repeated assessments are extremely important when using MMN as a neurophysiological predictor of coma emergence.

## 1. Introduction

Coma represents the most severe disruption in wakefulness and awareness that arises when cortical and brainstem pathways are damaged as a result of a catastrophic brain injury due to traumatic or non-traumatic causes (1). In comparison to other neurological conditions with impaired consciousness, the coma state usually resolves within days or a few weeks, and eventually evolves toward other states along the spectrum from full recovery to minimally conscious state (MCS), unresponsive wakefulness syndrome (UWS), or death (2). Since there is no assessment technique that can reliably detect any sign of inner awareness in comatose patients, the clinical evaluation typically focuses on detecting the level of functional impairment by using scores from traditional behavioral scales.

Recent guidelines provided by the American Academy of Neurology (AAN) and the European Academy of Neurology (EAN) recommend that patients in coma or other disorder of consciousness (DOC) should be diagnosed by using a multimodal approach including a comprehensive behavioral assessment along with advanced electroencephalography or functional neuroimaging, particularly in patients without command following abilities (3, 4). Although not available in all hospitals, these techniques seem promising for increasing diagnostic accuracy and refining the current model of assessing consciousness and cognitive function. These neurofunctional tests might therefore help to reduce the ~40% misdiagnosis rate found in patients who emerge from coma into other states including MCS or locked-in that are often classified as UWS despite a rigorous clinical assessment (5, 6).

It is even more challenging to predict how one will progress while still in a coma state. In the acute stage post-injury, the decisions made in intensive care have a major impact on patient survival and outcome (7, 8). Critically ill comatose patients may be too unstable clinically to be transferred from the intensive care unit (ICU) for functional neuroimaging assessments (9). The point-of-care aspect of the electroencephalogram (EEG) makes it a suitable tool for bedside assessment. One of the most commonly used approaches when investigating cognitive function and coma outcome are event-related potentials (ERPs), which are time-locked electrophysiological brain responses elicited typically by auditory, visual or tactile stimuli. Particularly, the mismatch negativity (MMN) has been considered a useful predictor of emergence from coma (10–13), and a key early biomarker in the information processing hierarchy leading up to conscious perception (14).

The auditory MMN (15) is a neural response to any discriminable change in a repetitive sequence of otherwise identical sounds. The MMN occurs within the time span of sensory memory and is considered independent of volitional attention and task performance. It is usually recorded within the “auditory oddball paradigm” in which repeated identical “standard” stimuli are interspersed with infrequent or “deviant” stimuli. The MMN has long been considered as an automatic pre-attentive ERP component, since it can be elicited in coma, during particular sleep stages and in the absence of behavioral discrimination ability (16). This claim has been refuted, however, due to a growing body of research showing systematic modulation of MMN amplitude with attention to the stimuli [see (17) for a review]. The frontal contribution to the attentional network, of which the MMN is part, results in further processing focused on the deviant stimulus. Accordingly, the MMN is often followed by the P3a component that indexes involuntary attention switch or reorientation to the deviants initiated by the MMN generation (15, 18, 19).

A different neurophysiological interpretation has been proposed that raises the question of whether the MMN is an indicator of “partial awareness” in the absence of overt behavior (20, 21). Using a masking experimental task, Dykstra and Gutschalk (20) demonstrated that the MMN is observed only when listeners were aware of the standard stream prior to the onset of the deviant. This approach better explains the presence of MMNs during states of behavioral unconsciousness such as sleep, coma, and other DOC (i.e., MCS and UWS), where a certain level of awareness of sensory stimuli is more likely than the ability to “attend” to them. Moreover, the MMN appears to be abolished during deep sedation-induced unconsciousness but returns as patients recover from anesthesia (22, 23). Although dissociating attention from consciousness is extremely difficult, a large body of evidence demonstrates that the MMN is highly correlated with emergence from coma and recovery of consciousness; and this evidence suggests that the MMN may be one of the earliest indicators of partial awareness in such patients.

A pioneering study found that over 91% of comatose patients exhibiting the MMN returned to consciousness (i.e., indicating a high positive predictive value), and over 90% of those who did not show MMN were considered as non-awake patients^1^ (i.e., reflecting high specificity). However, only about 30% of patients who emerged from coma showed a MMN, suggesting poor sensitivity (24). Subsequent studies confirmed the strong specificity and positive predictive value of MMN (25), but the sensitivity rate continued to be low, reaching values of about 56% when functional outcome was assessed 1 month after MMN recordings (26) and 32% when it was evaluated 12 months after coma onset (27). This low sensitivity constitutes a problem for prognosis; while it is possible to state with some confidence that emergence from coma is highly likely once MMN is present, patients who do not show the response can also emerge. Nevertheless, failure to detect the MMN should be interpreted with caution and not be taken as a definitive “absence of response.” It is possible that different analysis methods may be better at detecting the MMN. A recent study using machine learning showed that the MMN waxes and wanes in comatose patients when assessed across 24 h (13). This cycling pattern of presence/absence was postulated to be the predominant explanation for the low sensitivity rates reported in previous studies. These findings suggest that the MMN should no longer be sought in single-block recording sessions as has been done traditionally for decades. A testing session should be repeated several times, over the course of hours or longitudinally across different days to increase the chances of detecting the MMN and thus improving its sensitivity and relevance to patient care.

This approach of repeated or extended testing must also apply when evaluating healthy control subjects for comparisons, since there is evidence -albeit to a lesser extent- that not all healthy individuals exhibit the MMN in a single first assessment (28). Single-subject analyses can indeed provide useful information that is obscured or simply not available in the average responses observed across a group of control participants. This is particularly important for DOC research, since to interpret patient data accurately in clinical settings, it is crucial to identify reliable ERPs at a single subject-level, but also to design experimental paradigms able to elicit such responses.

In the present study, as part of an ongoing longitudinal study (29), we investigate the auditory MMN responses in healthy controls recorded over a 12-h period that were then analyzed at both the group and single-subject levels. We also report the results of three cases of coma patients whose MMNs were assessed repeatedly over a 24-h period at two different time points. We sought to investigate if the MMN exhibits fluctuations over time in healthy, fully-conscious states of awareness, or whether such waxing/waning is a specific feature of coma.

## 2. Materials and methods

### 2.1. Healthy controls and comatose patients

In order to characterize typical ERP responses across a period of up to 12 h during full conscious awareness and to obtain a baseline for the experimental paradigm, 17 healthy control participants (14 females) were recruited. Participants were aged between 19 and 56 years old (mean = 29.64, SD = 11.73) and had no history of neuropsychiatric disorders, alcohol/drug abuse, head trauma, or known hearing impairment. Participants were paid $15/h up to a maximum of $180 at the end of the study period. The study was approved by the Hamilton Integrated Research Ethics Board (HiREB; project number 4840).

Continuous EEG/ERP data were collected over the course of 24 h from three female comatose patients. All were assessed over the course of 2 days in either the ICU or the neurological Step-Down Unit at the Hamilton General Hospital, and were classified as being in a comatose state with Glasgow Coma Scale (GCS) scores <8 at the first day of recording. Patients 1 and 2 had neurosurgical complications as their coma etiology, while Patient 3 had a traumatic brain injury following a road traffic accident (see Table 1). Patients were off sedative medications during the EEG recordings. This included anesthetic agents such as propofol or small doses of benzodiazepines (e.g., midazolam) that were withheld for a minimum of 2 h prior testing. Exclusion criteria included seizure or epileptiform activity, known hearing impairment, medically induced coma, severe liver, and renal failure.

**Table 1:** Patient demographics and clinical information.

**Patient**	**Sex**	**Age**	**Etiology**	**Testing Day**	**State**	**Days since coma onset**	**GCS (E,V,M)**	**FOUR (E,M,B,R)**	**Blocks recorded**
1	F	41	Neurosurgery	0	Coma	20	5 (1,1,3)	6 (0,1,4,1)	8
				3	Coma/UWS	23	6 (4,1,1)	8 (3,0,4,1)	10
2	F	51	Neurosurgery	0	Coma	8	5 (1,1,3)	5 (0,1,4,0)	10
				3	Awakening	11	9 (4,1,4)	9 (3,1,4,1)	2
3	F	43	Trauma	0	Coma	13	4 (1,1,2)	5 (0,1,4,0)	10
				3	Coma	16	7 (2,1,4)	5 (0,1,4,0)	6

GCS, Glasgow Coma Scale, which is the sum of E (Eye opening) + V (Verbal response) + M (Motor response) scores; FOUR, Full Outline of Unresponsive score that has four components—E, Eye response; M, Motor response; B, Brainstem reflexes; R, Respiration.

### 2.2. Behavioral coma assessments

Diagnosis of coma and outcome were assessed by the GCS and the Glasgow Outcome Scale (GOS) (30), respectively. In general, the GCS is usually applied to determine severity of coma and includes three aspects of behavioral responsiveness: eye opening, verbal, and motor responses. The GOS globally rates the functional outcome for patient states into one of five categories: dead, vegetative state (VS; currently known as UWS), severe disability, moderate disability, or good recovery.

In addition, we used the Full Outline of UnResponsiveness score (FOUR). The FOUR includes assessment of eye movements and brainstem reflexes, which are unavailable with the GCS. It reduces misdiagnosis of locked-in syndrome and MCS by including assessment of eye movement, and helps to distinguish between comatose and recovering patients (4).

### 2.3. Stimuli and procedure

The MMN was recorded in an auditory three-deviant oddball paradigm (31), as part of a modified implementation of an ongoing study (29). Two thousand and four hundred tones at a regular 450-ms stimulus onset asynchrony (SOA) were recorded. The sequence comprised 82% standard tones (50 ms, 1,000 Hz, 80 dB) sound pressure level (SPL) and three types of deviant tones (6% each): a duration deviant (125 ms), a frequency deviant (1,200 Hz), and an intensity deviant (90 dB SPL). Auditory stimuli were delivered through noise-canceling insert earphones (Etymotic ER-1) using Presentation software (Neurobehavioral Systems, Inc.). This was a passive task that lasted ~25 min, with no behavioral responses required.

Healthy control subjects participated in several EEG/ERP tasks, that were repeated for a test day of up to 12 h. This schedule produced between three and five MMN recording blocks. Sufficient breaks were provided to the control subjects during the day to minimize movement artifacts and fatigue. Patients were tested in two recording sessions conducted 3 days apart, denoted as day 0 and day 3, respectively. Each recording session lasted up to 24 h with all testing done at the patient's bedside. Each test day comprised the same EEG/ERP protocol used in controls, including the MMN paradigm with resting state periods (10 min each) between each task. Behavioral scales were applied at the beginning of each testing day, before the EEG/ERP recordings. According to our protocol (29), if patients were emerging (i.e., awakening, eyes opening) from coma, only two blocks of the oddball paradigm were recorded.

### 2.4. EEG recording and preprocessing

EEG was recorded online with a bandpass of 0.01–100 Hz and sampled at 512 Hz. For healthy controls and one comatose patient, the electrodes were placed on the scalp according to the extended 10/20 system using a 64-electrode cap. A reduced number of 11 electrodes (F3, Fz, F4, C3, Cz, C4, P3, Pz, P4, T7, T8) were used following the same 10/20 system in two patients (Patient 1 and 2) due to surgical incisions and external ventricular drains (EVD). For all controls and patients, vertical and horizontal electrooculogram (EOG) signals were monitored by electrodes placed above and over the outer canthus of the left eye, and reference electrodes were located bilaterally at the mastoids.

Data pre-processing was conducted offline (Brain Products Inc.). All recordings were filtered with a bandpass of 0.1–30 Hz. Epochs containing non-ocular artifacts (e.g., muscle activity, movements) were removed. Ocular artifacts were corrected using the Independent Component Analysis (ICA) transformation (32). EEG trials were separated and segmented by stimulus type from 100 ms pre-stimulus to 600 ms post-stimulus and baseline corrected (−100 to 0 ms). These segments were averaged together per condition (i.e., stimulus type) for each block and subject or patient.

### 2.5. Statistical analysis

In addition to visual identification of the averaged ERP components, two main statistical methods were used to detect the presence of these components for each recorded MMN block at both the group level and the single-subject level in healthy controls.

#### 2.5.1. Permutation *t*-test

Permutation testing comes from a classical inference approach that relies on the use of null hypothesis significance testing, featuring the *p*-value as an indication of whether this hypothesis is probably true or false. The *p*-value could be derived from comparison to a Monte Carlo estimate of a permutation distribution, generated by randomly exchanging the trials from different conditions. In comparison to other conventional statistical tests, the permutation test seems to be preferred because of its greater statistical power, reliability for small samples and independence from any assumptions related to normal distribution of data and homogeneity of variances that are required when using parametric tests such as *t*-tests and analysis of variance (ANOVA) (33).

Here, one-tailed serial permutation *t*-tests were performed over a mean of six frontocentral electrodes (F3, Fz, F4, C3, Cz, C4) at each time point to find the intervals where the deviant condition was significantly more negative (e.g., a MMN component) or positive (e.g., a P3a component) compared to the standard condition. For group-level analyses, dependent samples permutation-*t* testing was performed across individual-averaged ERPs for the entire epoch (−100 to 600 ms). Maximum effect sizes (Cohen's *d*) were calculated over 50 and 100 ms periods surrounding the peak latency, which was automatically detected as the most negative or positive peak within each component window of interest respectively (MMN: 80–230 ms and P3a: 250–350 ms). For single-subject analyses, independent samples permutation *t*-tests were conducted across trials/epochs from each subject. For both analyses, the number of permutations was set to 1,000, the *p*-values were corrected using the *T*_*max*_ statistic for multiple comparisons.

#### 2.5.2. Bayesian analysis

Bayesian hypothesis testing presents an attractive alternative to *p*-values, which have been criticized extensively in the literature (34–36). This analysis is powerful as it provides weights of evidence for or against both the alternative and null hypotheses. Here, the strength of the evidence in favor of the alternative hypothesis *H*1 (difference between standard and deviants) over null hypothesis *H*0 (no difference), was quantified by Bayes factors (*BF*_10_). Maximum Bayes factors were calculated over the previous time periods surrounding the peak latency for each component of interest. Traditional interpretations of cut-offs (37) were modified by Lee and Wagenmakers (38), resulting in the following ranges: 1–3: anecdotal evidence, 3–10: moderate; 10–30: strong; 30–100: very strong, and >100: extreme. Analyses were done in Matlab, version R2020a (MathWorks Inc., USA), using a function from the FieldTrip toolbox for electrophysiological data analysis, which supports both unpaired and paired designs and assumes flat priors (39).

Additionally, in order to compare the MMN responses elicited by each deviant and determine whether there were habituation effects over time in the control group, we conducted a repeated measures analysis of variance (ANOVA) with deviant type (duration, frequency, and intensity) and block (1–5 blocks recorded over time) as within-subject factors at a cluster of six frontocentral electrodes (described above) with amplitude as the dependent variable. Mean amplitudes were exported in a ±50 ms window surrounding the group average peak: 180–230 ms for duration MMN and 80–130 ms for frequency and intensity MMN. When statistically significant differences were found, a Bonferroni *post-hoc* test was conducted for multiple comparisons. A Geisser and Greenhouse test for sphericity correction was used when appropriate (40). This analysis was conducted using JASP software (version 0.14.1).

For the comatose patients, a similar procedure as outlined above (visual inspection, serial permutation *t*-test, and Bayesian analysis) was performed at the single-subject level for every recorded MMN block.

## 3. Results

### 3.1. MMN in controls: From group-level to single-subject analysis

**Figure 1:**
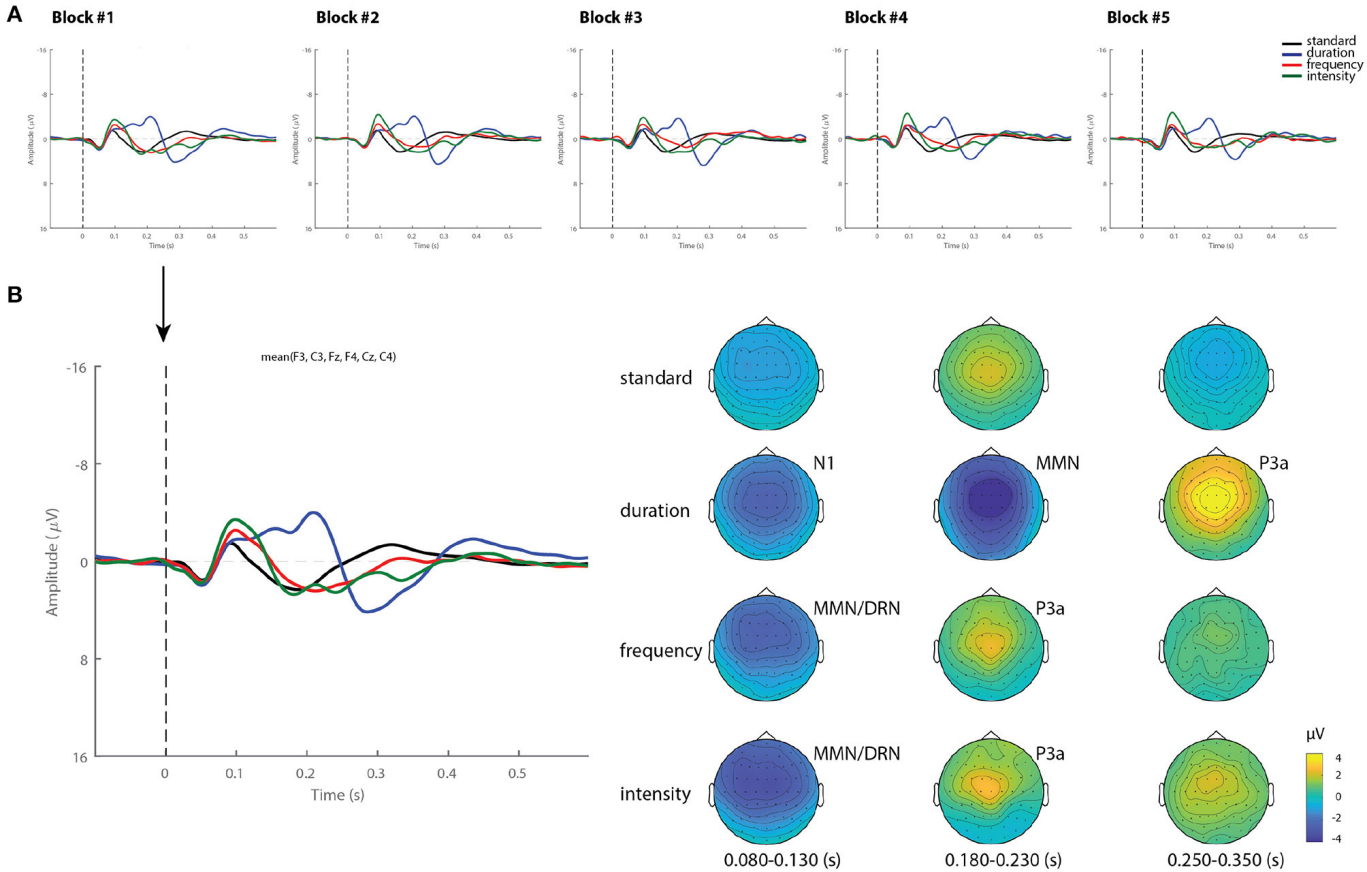
Grand-average ERPs and topography in healthy controls. **(A)** Grand-average ERP for each stimuli (standard, duration, frequency, and intensity) across blocks. **(B)** Example of ERP waveforms and scalp topographical maps of Block 1 averaged over 80–130, 180–230, and 250–350 ms time intervals.

Figure 1A shows the grand-average ERPs over a mean of six frontocentral electrodes (F3, Fz, F3, C3, Cz, C4), corresponding to standard and deviant stimuli (duration, frequency, and intensity) for each block recorded over a 12 h period. As can be observed, the waveforms from all blocks were extremely similar. Figure 1B displays the ERP waveforms from Block 1 as an example, and its corresponding topographical maps averaged over 80–130, 180–230, and 250–350 ms time intervals. Frequency and intensity deviants elicited a negative component peaking between 80 and 130 ms, which represents a spatial-temporal summation of both N1 and MMN components, often called deviant-related negativity (DRN) (41). This was followed by a frontocentral positivity (P3a), which peaked later between 180 and 230 ms. The duration deviant elicited three dissociated components: the N1 peaking at 150 ms, a MMN with maximum amplitude between 180 and 230 ms and a P3a component with maximum amplitude between 250 and 350 ms.

**Figure 2:**
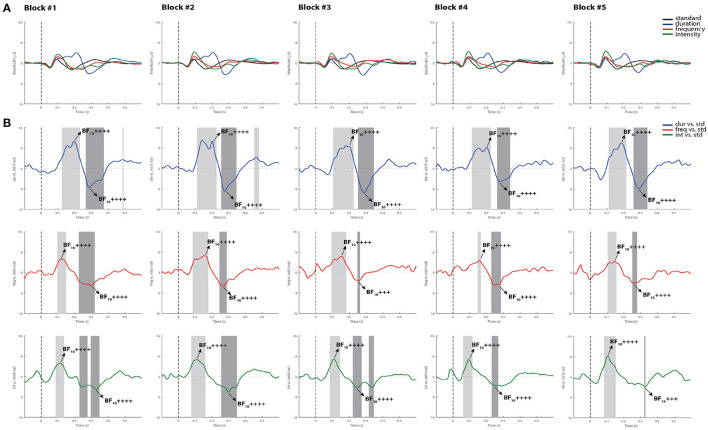
Grand-average ERPs and statistical findings in healthy controls. **(A)** Grand-average ERPs across blocks. **(B)** Time course of the difference between deviants and standard stimuli expressed in units of *t*-values. Significant intervals for negative components are denoted by a light gray area, and positive components are denoted by a dark gray area. Black arrows show the latency of maximum Bayes factors and the strength of evidence for *H*1: +, anecdotal; ++, moderate; +++, strong, ++++, very strong to extreme.

Figure 2B summarizes the statistical findings at the group level in healthy controls. As can be observed, both the MMN and the P3a components were observed in all blocks, and reliably detected by using permutations *t*-test (*p* < 0.05) and Bayes factor analysis.

For the MMN component, the Cohen's d computed from the permutation *t*-tests were averaged across all five blocks, reaching values of 1.28 for the duration deviant, 0.79 for frequency, and 1.15 for intensity, indicating a very large, a medium and a large effect size, respectively, according to (42). For the P3a, the averaged Cohen's *d* indicated a huge effect size for duration (2.25), and a very large effect size for both frequency (1.39) and intensity (1.51). Maximum Bayes factors computed at the time window of interest for each component mostly revealed very strong to extreme evidence for our hypothesis of significant difference between the deviant and standard stimuli in all blocks (see Cohen's *d* and Bayes factors for each recorded block in Supplementary Table 1).

**Figure 3:**
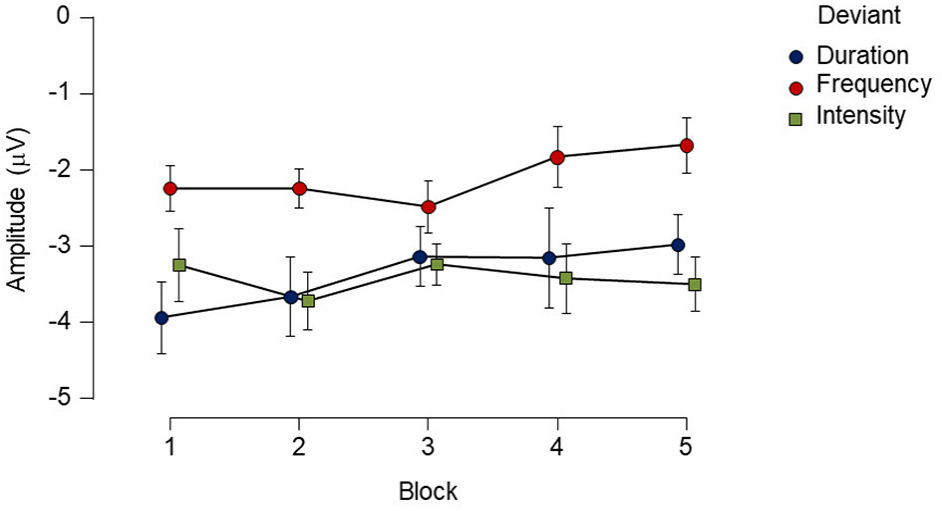
Mean amplitude and standard errors (SE) of each deviant type for each block recorded over a 12-h period in the healthy control group. While there was main effect of deviant type, no reliable main effect of block or interaction was found. Points represent mean amplitude from each deviant type. Vertical extended lines indicate the standard error intervals.

When differences between deviants and habituation effects were evaluated within the group, the repeated-measures ANOVA analysis showed a main effect for deviant type [*F*_(2, 24)_ = 7.13, *p* < 0.05, $\eta_{p}^{2}=0.37$]. A Bonferroni *post-hoc* test averaged over the levels of blocks revealed differences between duration and frequency deviants with a mean difference of −1.27 μV (*p* < 0.05), and between intensity and frequency with a mean difference of 1.33 μV (*p* < 0.01). No significant main effect was found for block [*F*_(4, 48)_ = 0.71, *p* = 0.52, $\eta_{p}^{2}=0.05$], and the deviant type x block interaction also failed to reach significance [*F*_(8,96)_ = 0.84, *p* = 0.47, $\eta_{p}^{2}=0.06$; see Figure 3].

Single-subject analysis showed that both MMN and P3a components elicited by the duration deviant were significantly detected across all blocks in all subjects by using both permutation *t*-test and Bayesian analysis. The serial permutation *t*-test showed that 3 out of 17 subjects did not exhibit significant MMNs to the frequency deviant in any of the recorded blocks, and one subject to the intensity deviant. Bayesian analysis showed evidence in favor of the presence of MMN responses to frequency and intensity deviants in all subjects in at least one block, confirming the visual inspection. Tables 2, 3 summarize the proportion of control subjects exhibiting MMN and P3a responses, respectively, for each recorded block using the three methods adopted in the present study. Notice (in N column) that not all subjects performed all blocks, but regardless the sample size, the duration deviant still elicited the most reliable responses, as can also be observed in Figure 2.

**Table 2:** Proportion of healthy controls showing evidence of MMN in each block.

		**Duration**			**Frequency**			**Intensity**		
**Block**	***N***	**Visual**	**Perm. test**	**Bayes**	**Visual**	**Perm. test**	**Bayes**	**Visual**	**Perm. test**	**Bayes**
1	17	1.00	1.00	1.00	0.88	0.47	0.65	1.00	0.71	0.82
2	17	1.00	1.00	1.00	0.82	0.53	0.82	0.94	0.76	0.94
3	17	1.00	1.00	1.00	0.88	0.47	0.88	0.88	0.53	0.82
4	14	1.00	1.00	1.00	0.86	0.50	0.71	0.93	0.57	0.93
5	13	1.00	1.00	1.00	0.84	0.31	0.71	1.00	0.46	1.00

**Table 3:** Proportion of healthy controls showing evidence of P3a in each block.

		**Duration**			**Frequency**			**Intensity**		
**Block**	***N***	**Visual**	**Perm. test**	**Bayes**	**Visual**	**Perm. test**	**Bayes**	**Visual**	**Perm. test**	**Bayes**
1	17	1.00	0.82	0.94	0.82	0.47	0.71	0.88	0.71	0.82
2	17	1.00	0.88	1.00	0.53	0.41	0.47	0.88	0.76	0.82
3	17	1.00	0.88	1.00	0.53	0.18	0.53	0.82	0.71	0.76
4	14	1.00	0.79	0.93	0.57	0.21	0.50	0.71	0.71	0.64
5	13	1.00	0.85	1.00	0.54	0.38	0.38	0.69	0.46	0.54

**Figure 4:**
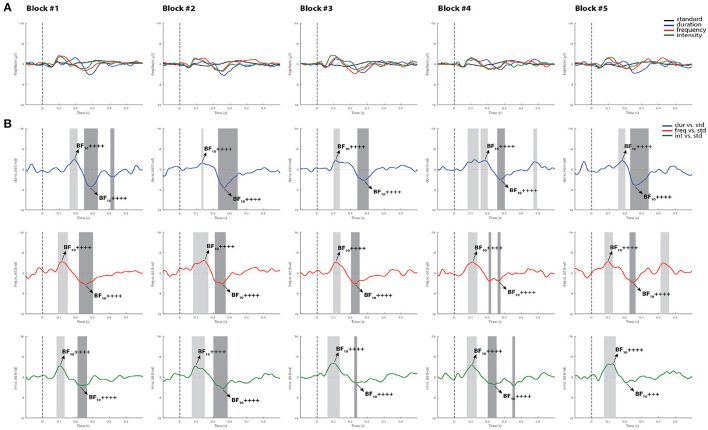
Individual ERPs and statistical findings of a representative control subject with the highest MMN detection rate. **(A)** Individual ERPs across blocks. **(B)** Time course of the difference between deviants and standard stimuli expressed in units of *t*-values. Significant intervals for negative components are denoted by a light gray area, and those for positive components are denoted by a dark gray area. Black arrows show the latency of maximum Bayes factors and the strength of evidence for *H*1: +, anecdotal; ++, moderate; +++, strong; ++++, very strong to extreme.

**Figure 5:**
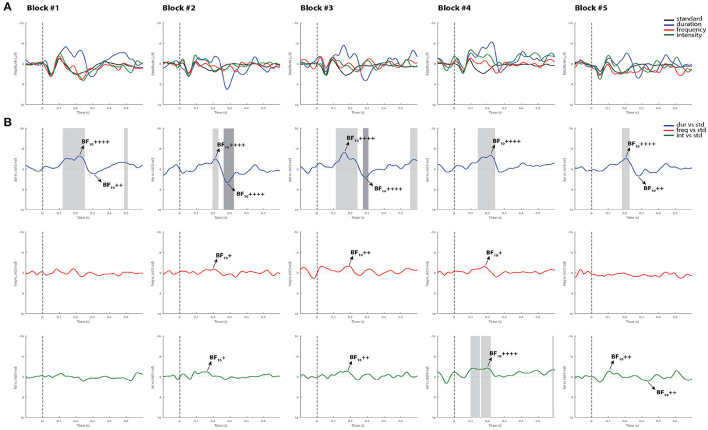
Individual ERPs and statistical findings of a representative control subject with the lowest MMN detection rate. **(A)** Individual ERPs across blocks. **(B)** Time course of the difference between deviants and standard stimuli expressed in units of *t*-values. Significant intervals for negative components are denoted by a light gray area, and those for positive components are denoted by a dark gray area. Black arrows show the latency of maximum Bayes factors and the strength of evidence for *H*1: +, anecdotal; ++, moderate; +++, strong; ++++, very strong to extreme.

Figures 4, 5 display the results from two representative control subjects, showing the highest and lowest ERP detection rates, respectively. As shown in these examples, the first control subject (see Figure 4) had the highest ERP detection rate, exhibiting significant MMN intervals for each deviant sound in all recorded blocks when performing all methods of analysis. A reliable P3a response was also found in most of the blocks and deviant conditions, except in the fifth block for the intensity deviant. The second control subject, who had the lowest ERP detection rate (see Figure 5) exhibited a significant duration MMN in all recorded blocks with all methods, but the permutation *t*-test failed to capture a significant MMN in all blocks for the frequency deviant and in fourth blocks for the intensity deviant. The Bayesian analysis confirmed the visual inspection method by showing anecdotal or moderate evidence for the presence of a MMN in three blocks for frequency and intensity deviants.

### 3.2. MMN in coma: Case reports

#### 3.2.1. Patient 1: From step-down unit to palliative care

Patient 1 was a 41-year-old woman who was admitted to the step-down unit 20 days prior to our assessment. The patient had a history of multiple re-resections of a left frontotemporal oligodendroglioma. She was admitted to the neurosurgery operating room for surgical repair of cerebrospinal fluid (CSF) leak, which required reopening of left frontal subdural craniotomy for a lumbar drain and a subsequent right ventriculoperitoneal (VP) shunt. A right frontal EVD was inserted after a shunt infection and an intracranial abscess resection was performed at the previous surgical site. At the time of the first assessment (day 0), the patient had a GCS score of 5. A summary of the MMN results for this patient are given in Table 4. Out of eight recorded blocks collected on the first day (day 0), a reliable MMN to all deviants was detected in one block (block 4) by using all methods of analysis, and in an additional block (block 6) for the intensity deviant. Also, a significant P3a component was detected in two blocks (blocks 4 and 6) for the intensity deviant in Supplementary Table 2 for the summary of the P3a results. On day 3, the patient had spontaneously opened her right eye, which remained persistently halfway open requiring artificial tears or eye pads to prevent corneal abrasions. The patient, however, did not fixate to stimuli or track (see FOUR score in Table 1). Confirmed by the three selected analysis methods, the patient had a reliable MMN response in 4 out 10 recorded blocks for the duration deviant (blocks 2, 3, 8, and 10), in block 8 for frequency and in block 2 for intensity. A P3a component, was also detected in 6 blocks for the intensity deviant (blocks 2, 3, 6, 8, 9, and 10), in 4 blocks for duration (blocks 2, 6, 9, and 10) and in two blocks for the frequency deviant (blocks 6 and 9; see Supplementary Table 2).

**Table 4:** Summary of the MMN results in Patient 1.

**Day 0**				**Duration**			**Frequency**			**Intensity**		
**GCS**	**FOUR**	**Block**	**Time**	**Visual**	**Perm. test**	**Bayes**	**Visual**	**Perm. test**	**Bayes**	**Visual**	**Perm. test**	**Bayes**
5	6	1	18:35 p.m.	+	−	+	+	−	−	−	−	−
		2	21:10 p.m.	+	−	+	+	−	+	+	−	+
		3	22:49 p.m.	−	−	−	−	−	−	−	−	−
		4	12:47 a.m.	+	+	++++	+	+	+++	+	+	++++
		5	06:26 a.m.	+	−	+	−	−	−	+	−	++
		6	07:58 a.m.	−	−	−	−	−	−	+	+	++++
		7	10:08 a.m.	+	−	−	+	−	−	+	−	−
		8	11:26 a.m.	+	−	++	+	−	−	+	−	++
**Day 3**				**Duration**			**Frequency**			**Intensity**		
**GCS**	**FOUR**	**Block**	**Time**	**Visual**	**Perm. test**	**Bayes**	**Visual**	**Perm. test**	**Bayes**	**Visual**	**Perm. test**	**Bayes**
6	8	1	18:20 p.m.	−	−	−	+	−	−	+	−	−
		2	20:29 p.m.	+	+	++++	+	−	+++	+	+	++++
		3	21:15 p.m.	+	+	+++	−	−	−	−	−	−
		4	23:14 p.m.	+	−	−	−	−	−	−	−	−
		5	01:05 a.m.	+	−	+	+	−	+	−	−	−
		6	03:13 a.m.	+	−	−	−	−	−	−	−	−
		7	05:22 a.m.	−	−	−	−	−	−	−	−	−
		8	06:09 a.m.	+	+	+++	+	+	++	+	−	−
		9	07:56 a.m.	−	−	−	+	−	−	+	−	+
		10	10:11 a.m.	+	+	++	−	−	−	+	−	+
**OUTCOME: Death (withdrawal of life support)**												

+ indicates a positive result, − a negative result. For Bayes column, +, anecdotal evidence; ++, moderate evidence; +++, strong evidence; ++++, very strong to extreme evidence.

After a few days of the EEG assessment, the patient's clinical condition worsened. Active care was withdrawn while maintaining comfort measures. The patient subsequently died.

#### 3.2.2. Patient 2: From coma to awakening in intensive care

Patient 2 was a 53-year-old woman admitted to the ICU, deeply unconscious after a cystoperitoneal shunt malfunctioning that required neurosurgery. She had a history of meningioma resection from the right posterior cranial fossa, complicated by meningitis, CSF leak, and debridement surgeries.

A summary of results for this patient are given in Table 5. On day 0, the patient showed a significant MMN in 2 out of 10 recorded blocks (blocks 9 and 10) for the duration deviant according to all selected methods, and in block 10 for the frequency deviant. A significant P3a component was detected in 2 blocks for the intensity deviant.

**Table 5:** Summary of the MMN results in Patient 2.

**Day 0**				**Duration**			**Frequency**			**Intensity**		
**GCS**	**FOUR**	**Block**	**Time**	**Visual**	**Perm. test**	**Bayes**	**Visual**	**Perm. test**	**Bayes**	**Visual**	**Perm. test**	**Bayes**
5	5	1	21:15 p.m.	+	−	−	+	−	−	−	−	−
		2	12:14 a.m.	−	−	−	+	−	+	+	−	−
		3	02:24 a.m.	−	−	−	−	−	−	−	−	−
		4	4:51 a.m.	+	−	+	−	−	−	−	−	−
		5	06:50 a.m.	+	−	−	+	−	++	+	−	+
		6	08:00 a.m.	−	−	−	−	−	−	+	−	−
		7	10:09 a.m.	−	−	−	+	−	−	−	−	−
		8	11:48 a.m.	−	−	−	−	−	−	−	−	−
		9	12:28 a.m.	+	+	++	−	−	−	+	−	+
		10	02:37 p.m.	+	+	+++	+	+	++	−	−	−
**Day 3**				**Duration**			**Frequency**			**Intensity**		
**GCS**	**FOUR**	**Block**	**Time**	**Visual**	**Perm. test**	**Bayes**	**Visual**	**Perm. test**	**Bayes**	**Visual**	**Perm. test**	**Bayes**
9	9	1	18:34 p.m.	+	+	++++	+	−	++	+	−	−
		2	20:48 p.m.	+	−	−	+	−	++	+	−	−
**OUTCOME: Good recovery**												

+ indicates a positive result, − a negative result. For Bayes column, +, anecdotal evidence; ++, moderate evidence; +++, strong evidence; ++++, very strong to extreme evidence.

The second recording, denoted as day 3, included only two blocks of the MMN paradigm, since the patient exhibited behavioral signals of emerging from coma state as shown in GCS and FOUR scales in Table 1. The three methods confirmed the presence of a MMN only for the duration deviant in one of the recorded blocks and a P3a response in both blocks. (See summary of the P3a results in Supplementary Table 3).

This patient was subsequently transferred to the neurosurgery inpatient unit where she was awake, oriented and talking. After a year, the patient had resumed her normal life with minor neurological deficits, which is congruent with a good recovery outcome.

#### 3.2.3. Patient 3: Coma following multisystem trauma

This was a 43-year-old patient included in the study 13 days post hospital admission for severe multisystem trauma after being involved in a road vehicle accident. On arrival to the ICU, she was intubated and sedated with a GCS of 3. Computed tomography scans revealed bilateral subarachnoid hemorrhage, with no herniation as well as diffuse axonal injury. Her GCS was 4 and 7 during the first and second EEG recordings, respectively (see Table 1).

On day 0, the patient showed a MMN confirmed by the three methods in block 8 for the duration deviant and in block 10 for the frequency deviant (block 10). A P3a component was reliably detected for the frequency deviant in block 8 and for the intensity deviant in the blocks 7 and 8 with all the methods. The presence of this component was confirmed in other blocks by two methods (visual inspection and Bayesian analysis).

On day 3, only a duration MMN was confirmed by visual and Bayesian analysis in 3 blocks (see Table 6). A P3a response to duration and frequency deviants was confirmed by all methods in one block (block 2; see summary of the P3a results in Supplementary Table 4).

**Table 6:** Summary of the MMN results in Patient 3.

**Day 0**				**Duration**			**Frequency**			**Intensity**		
**GCS**	**FOUR**	**Block**	**Time**	**Visual**	**Perm. test**	**Bayes**	**Visual**	**Perm. test**	**Bayes**	**Visual**	**Perm. test**	**Bayes**
4	5	1	14:52 p.m.	+	−	−	−	−	−	−	−	−
		2	16:44 p.m.	−	−	−	−	−	−	−	−	−
		3	18:46 p.m.	−	−	−	−	−	−	−	−	−
		4	20:55 p.m.	+	−	+	+	−	+++	−	−	−
		5	21:36 p.m.	−	−	−	+	−	+	+	−	+
		6	23:25 p.m.	+	−	+	−	−	−	+	−	−
		7	01:34 a.m.	+	−	+	+	−	+	+	−	+
		8	02:50 a.m.	+	+	++	−	−	−	+	−	+
		9	05:24 a.m.	−	−	−	+	−	−	+	−	−
		10	07:09 a.m.	+	−	−	+	+	+++	+	−	+
**Day 3**				**Duration**			**Frequency**			**Intensity**		
**GCS**	**FOUR**	**Block**	**Time**	**Visual**	**Perm. test**	**Bayes**	**Visual**	**Perm. test**	**Bayes**	**Visual**	**Perm. test**	**Bayes**
7	5	1	20:46 p.m.	−	−	−	−	−	−	+	−	−
		2	21:45 p.m.	−	−	−	−	−	−	−	−	−
		3	23:29 p.m.	−	−	−	−	−	−	−	−	−
		4	01:38 a.m.	+	−	+	−	−	−	+	−	+
		5	04:33 a.m.	+	−	+	+	−	−	−	−	−
		6	06:21 a.m.	+	−	+	+	−	−	−	−	−
**OUTCOME: UWS**												

+ indicates a positive result, − a negative result. For Bayes column, +, anecdotal evidence; ++, moderate evidence; +++, strong evidence; ++++, very strong to extreme evidence; UWS, unresponsive wakefulness syndrome.

This patient was transferred to another hospital. Based on her records, the patient remained dependent on the ventilator and the tracheotomy by the time of discharge. She was withdrawing and flexing to pain, and would occasionally open her left eye spontaneously, but not to voice or pain and would not track. She then was transferred to chronic care, after being diagnosed as a VS/UWS patient.

All individual ERPs and statistical findings from all comatose patients are displayed in Supplementary Figures 1–10.

## 4. Discussion

This is the first study to continuously track MMN responses in healthy controls over an extended period of time. In this study we tested controls for a maximum period of 12 h as part of our ongoing EEG/ERP project to predict coma emergence and eventual functional outcome. The detection rate of the MMN was assessed over time at both the group and single-subject level using three different methods: traditional visual inspection of the averaged ERPs, permutation *t*-test and Bayesian analysis. We also provided preliminary evidence of the utility of monitoring auditory deviance detection in three comatose patients over a 24 h-period at two time points to predict coma outcome. We addressed the question of whether short-term fluctuations in MMN detectability may occur during full conscious awareness or is rather a feature of coma state (13); a finding that would have implications for prognostics of the timing of coma emergence and the clinical state at emergence. In turn, knowledge of the clinical state (i.e., UWS/VS, MCS, Locked-in) would encourage extended assessment of the cognitive state at emergence relevant to future rehabilitation efforts.

### 4.1. Tracking MMN in full conscious awareness

Our results showed that the MMN can be elicited and reliably detected over the course of 12 h in healthy control subjects at the group level. Serial permutation *t*-tests applied on a within-group design were able to capture significant differences between the three types of deviants (i.e., duration, frequency, intensity) and standard stimuli in both MMN and P3a components in all recorded blocks. Bayesian analysis confirmed these findings, by showing “very strong to extreme” evidence. Consistent with the present results, several studies have shown that reliable MMNs can be recorded from session to session in a group of subjects (43–45). In these studies of test-retest reliability, the MMN responses are usually obtained from different sessions or blocks separated by longer time-intervals (i.e., ~1 month or more). Using different methods, we found that the MMN can be consistently replicated and detected over intervals of hours in a continuous testing session.

Additionally, while differences in MMN mean amplitude were observed between deviant types within the group, showing smaller MMN responses to frequency deviants, no main effects of blocks recorded over time or interaction were found. In line with prior findings, where this multi-deviant oddball paradigm was first implemented, the duration and intensity MMNs from a group of controls were slightly larger in amplitude than those produced to the frequency deviant tone (31). Furthermore, the fact that no habituation effects of the MMN amplitude were found over time (see Figure 2), suggests that the detectability of this component is not compromised by the repetition of the oddball paradigm. Its replicability in such short periods of time at group-level highlights the use of appropriate stimuli, and the application of efficient recording procedures as a way to reduce the variability of the recorded responses (46).

On the other hand, the single-subject analysis in this study revealed that fluctuations in MMN detectability may be observed in some control subjects, depending on the deviant type, and the statistical method performed to confirm the presence of the component. As we have demonstrated and as illustrated in Table 2, the three methods showed a 100% detection rate of the MMN component for the duration deviant in all recorded blocks. However, the detection rate of subjects showing a reliable MMN response to frequency and intensity deviants at each recorded block was 65–71 and 82–100%, respectively with Bayesian analysis, and 31–47 and 46-71% with permutations. This latter test was more conservative, showing that 3 out of 17 subjects did not exhibit significant MMNs to the frequency deviant in any of the recorded blocks, and one subject failed to show any response to the intensity deviant in any block.

Consistent with our findings, another Bayesian approach was recently reported as the most liberal in comparison to other five statistical methods on its ability to detect ERP effects (47). This confirmation of neural responses through different statistical methods is especially important for coma research, as the ERPs from patients with brain injury at single-patient level exhibit notable differences in amplitude, latency, and scalp distribution in comparison to healthy controls, which makes the visual identification of ERP components extremely challenging. Visual inspection of ERPs remains fairly common practice (12), but as discussed extensively in the literature (48, 49), it can introduce significant bias. Besides, reliable visual inspection requires expertise that is not commonly available in the clinical setting (29). Also, the wide availability of statistical methods, revealing large discrepancies among them is a problem for clinicians. Gabriel and colleagues compared six different methods previously used in coma studies to identify the MMN responses, and showed that all six methods confirmed an MMN response in only 4 out of 27 subjects, but at least the combination of two methods confirmed the presence of MMN in all control subjects (49). One may argue that these methods greatly differ in their mathematical algorithms and answer fundamentally different questions, and therefore should not be expected to provide the same results.

As stated by Naccache et al. (50), it is an essential prerequisite of any functional brain test to show high sensitivity, especially to evaluate patients with brain injury and determine whether they will regain consciousness. The chosen methods should be able to detect the associated neural responses at the individual level in the majority of conscious controls. Otherwise, their use in patient populations could complicate the interpretation of the results. That is, if frequency stimuli elicit robust MMN responses at the group level in all recorded blocks, but such findings are not powerful at the single-subject level, then their use for clinical practice will be limited. Duration deviants, however, have been consistently reported throughout the clinical literature to be more sensitive than frequency deviants to measure neurological changes in various medical conditions (51–53) and as a consequence promise greater clinical utility.

### 4.2. Tracking MMN in coma

Data from three comatose patients revealed that the MMN component was present in at least one block per recording session by using the three methods of analysis, but fluctuated in detectability over the course of 24 h. This fluctuation was not observed in healthy controls for the duration deviant, which supports the hypothesis that the MMN responses may be present only transiently in the coma state (13). As expected, most of the blocks where the MMN was confirmed to be present, corresponded to the duration-deviant condition in two comatose patients (Patient 1 and 2). Multivariate analysis has demonstrated a better discrimination between standard and duration deviants than other types of deviant-stimuli in comatose patients (54), which is in line with the choice of using duration deviants in previous coma studies (11, 24).

**Figure 6:**
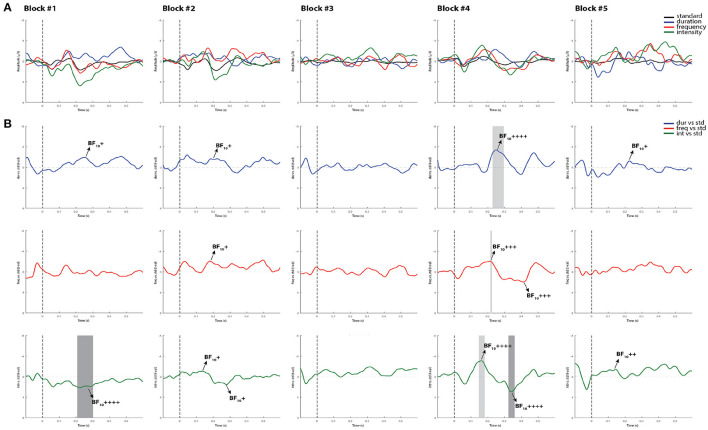
Individual ERPs and statistical findings of a coma patient (Patient 1) in the first five blocks on day 0. **(A)** Individual ERPs across blocks. **(B)** Time course of the difference between deviants and standard stimuli expressed in units of *t*-values. Significant intervals for negative components are denoted by a light gray area, and those for positive components are denoted by a dark gray area. Black arrows show the latency of maximum Bayes factors and the strength of evidence for *H*1: +, anecdotal; ++, moderate; +++, strong; ++++, very strong to extreme.

Similar to controls, the Bayesian analysis was more sensitive in capturing more blocks with reliable MMN and P3a responses than the permutation *t*-test in the comatose patients in both testing days. As displayed in the example in Figure 6, only one block (block 4) out of the first five recorded blocks showed significant MMN responses with all methods. In most cases where the ERP components were significantly detected by permutations, the Bayesian analysis served as a confirmatory test by indicating “moderate,” “strong,” or “very strong to extreme” evidence of the response. In the opposite direction, where the MMN was not significant by using permutations, but could be judged to be present through visual inspection, the Bayesian test would indicate in most cases weak or “anecdotal” evidence of response. It could be suggested that the visual inspection seems to be the most sensitive method here for detecting the MMN responses. However, this method is prone to bias and can often lead to false positives when not confirmed by other statistical analyses (49).

While the MMN has been reported to be a strong predictor of coma emergence and good functional outcome (25, 27, 55), we are fully aware that multiple factors might affect the patients' final outcome. For instance, multiple systemic complications mostly associated with infections are very likely to occur in critically ill patients, leading to further deterioration of their clinical status. Patient 1, for example, who showed more reliable MMN responses over time in comparison to the other patients, had the worst clinical outcome (i.e., death) after withdrawal of life support. In one of the first studies of Fischer and colleagues, three patients who had exhibited a MMN response failed to regain consciousness: one developed complications of neurosurgery, the second had organ failure complications and the third died of cardiac failure (24). Using a different MMN paradigm and a multivariate decoding algorithm, Tzovara's work also demonstrated intact auditory discrimination in comatose patients who eventually died (56). Consistent with these results, the robust presence of the MMN in Patient 1 is not surprising and could have indicated the patient's chance of emergence prior to unexpected complications.

One could argue that the “spontaneous” opening of the right eye in Patient 1 (without tracking or saccadic eye movements to stimuli) during the second recording (see Table 1), suggests that this patient was probably emerging and transitioning to UWS. Although we cannot rule out this possibility, it was recently claimed that some comatose patients, particularly those with supratentorial, infratentorial, or global brain insults, may defy the classical definition of coma (i.e., unarousable unresponsiveness with absent sleep cycles and closed eyes) by showing eye-opening (57). Coma with eye opening, according to these authors, differs from the UWS case in that it has a different clinical trajectory (tendency to worsening rather than stabilization) when accompanied by an absence of sleep-wake cycles. The authors also stated that behavioral scales, such as the Glasgow Coma Scale and the FOUR score, can yield misleading results and overly optimistic outcome estimations for comatose patients with eye opening. Unfortunately, we did not use other more sensitive diagnostic tools (e.g., Coma Recovery Scale-Revised) to confirm whether the patient was in UWS after day 3.

The other two patients in the present study emerged from coma, but exhibited different functional outcomes. Patient 2 emerged 3 days following the first EEG assessment and after a year showed a positive functional outcome (good recovery). Patient 3, however, was transferred to a different hospital and then to a chronic care facility, with a diagnosis of VS/UWS. Regardless of the functional outcome, the MMN was present in at least a single recording occasion for each patient. Coupled with previous evidence of the link between MMN detection and coma emergence, we suspect that continuous electrophysiological monitoring of the MMN may be instrumental in achieving improvements in the sensitivity of coma emergence prediction.

In general, the variability in the presence of the MMN component in these patients may be explained by their brain injuries and fluctuations in responsiveness inherent to DOC. Severe brain damage may alter ERP amplitude and topography, and cause temporal delays and inter-trial variability in comatose patients as result of white matter impairments and cortical dysfunction (58). Perhaps physiological artifacts (e.g., increased slow wave activity) and the inherent environmental artifacts of the intensive care settings may have added extra noise to the signal for the MMN to be objectively detected across all blocks. More extensive data collection is necessary to clarify the mechanisms behind these fluctuations.

### 4.3. Limitations and ethical implications

Our small sample-size limits the generalization of our findings and requires further replication in future work. Nevertheless, we consider these results relevant and very promising as they can serve as a foundation upon which to develop monitoring techniques for detecting transient periods associated with partial consciousness in patients with severe brain injury. Although it is challenging to run extended EEG studies without frequent interruptions in ICU environments, the recording of multiple blocks of data per day in a larger population would be ideal for tracking the trajectory of patients and identify those with potential for recovery.

Our different analysis methods used non-identical information about the waveforms, such as the selection of the time points. For instance, the permutation *t*-test was applied to the whole ERP time window in order to identify the significant latency windows of the MMN and P3a components, whereas the Bayesian analysis was applied to narrower time windows of interest. This approach of doing Bayesian test *post-hoc* after convincing results are obtained with permutations seems methodologically unnecessary. However, given that permutation *t*-tests are fairly conservative and showed more evidence in favor of null effects in comatose patients, the Bayesian evidence, even “weak or anecdotal” may still be valuable for this clinical population.

Importantly, the medical team responsible for patient care were blind to our results, which were never used to influence any clinical decision for treatment or the maintenance/withdrawal of life-sustaining therapies. The presence or absence of MMN alone did not impact such decisions. However, in the not too distant future it is apparent that the MMN, in combination with other potential biomarkers, could help critical care teams improve coma prognosis by relying on objective evidence rather than “perceptions of unfavorable prognosis for meaningful neurologic recovery” (7, p. 1) in making decisions about withdrawal of life-support within days of admission [see also (8, 59–61)].

## 5. Conclusion

The results of the present study demonstrate that the MMN responses elicited by duration deviant stimuli are consistently detected over time in healthy controls, at both group and single-subject levels. This finding supports the use of the MMN elicited by duration deviants as a promising tool for monitoring brain functional changes in clinical settings. Preliminary findings in three acute coma patients, recorded over 24 h, provide further evidence that the MMN is present in coma, but can be transient (i.e., waxes and wanes) across hours. This highlights that regular and repeated assessments are extremely important for clinically-appropriate usage of the MMN as a neurophysiological predictor of coma emergence.

## Data availability statement

The original contributions presented in the study are included in the article/Supplementary material, further inquiries can be directed to the corresponding author.

## Ethics statement

The studies involving human participants were reviewed and approved by Hamilton Integrated Research Ethics Board (HiREB; project number 4840). The patients/participants provided their written informed consent to participate in this study.

## Author contributions

JC, RB, JR, AF-R, CH, and AH-D contributed to the conception of the study. C-YL and PT contributed to the organization of the study and recruitment of controls. AH-D analyzed the data and wrote the first draft of the manuscript. AH-D, RK, NP, and EB conducted the experiments. All authors reviewed and edited the manuscript. All authors contributed to the article and approved the submitted version.

## Footnotes

^1^Non-awake was operationally defined using Glasgow Outcome Scale (GOS) criteria, with GOS levels of 1 or 2 (death or vegetative state).
