# Supplementary material for: Tracking auditory mismatch negativity responses during full conscious state and coma
Source: Front Neurol. 2023 Mar 10;14:1111691. doi: 10.3389/fneur.2023.1111691 (PMC10036371; doi:10.3389/fneur.2023.1111691)
Supplement: Supplementary file 1 [file Data_Sheet_1.pdf]

Supplementary Material

Table S1. Effect size of permutation *t*-test (Cohen's d) and Bayes factors (BF10) from the healthy control group across blocks

		MMN		P3a	
**Deviant**	**Block**	**Cohen's d**	**BF10**	**Cohen's d**	**BF10**
Duration	1	1.29	> 100	2.02	> 100
	2	1.59	> 100	2.49	> 100
	3	1.43	> 100	2.48	> 100
	4	1.05	> 100	1.66	> 100
	5	1.04	> 100	2.62	> 100
Frequency	1	1.08	> 100	1.37	> 100
	2	0.70	> 100	1.58	> 100
	3	0.72	> 100	1.06	72.9
	4	0.76	> 100	1.51	> 100
	5	0.73	> 100	1.45	> 100
Intensity	1	1.12	> 100	1.42	> 100
	2	1.04	> 100	2.15	> 100
	3	1.26	> 100	1.38	> 100
	4	1.19	> 100	1.37	> 100
	5	1.13	> 100	1.24	50.41

Table S2. Summary of the P3a results in Patient 1. + indicates a positive result, - a negative result. For Bayes column, + anecdotal evidence; ++ moderate evidence; +++ strong evidence and ++++ very strong to extreme evidence

**DAY 0**				**DURATION**			**FREQUENCY**			**INTENSITY**		
**GCS**	**FOUR**	**Block**	**Time**	**Visual**	**Perm. test**	**Bayes**	**Visual**	**Perm.test**	**Bayes**	**Visual**	**Perm.test**	**Bayes**
5	6	1	18:35 PM	-	-	-	-	-	-	+	+	++++
		2	21:10 PM	+	-	-	-	-	-	+	-	+
		3	22:49 PM	-	-	-	-	-	-	-	-	-
		4	12:47 AM	-	-	-	+	-	+++	+	+	++++
		5	06:26 AM	+	-	-	-	-	-	-	-	-
		6	07:58 AM	+	-	+	-	-	-	+	-	-
		7	10:08 AM	-	-	-	-	-	-	-	-	-
		8	11:26 AM	+	-	++	+	-	-	-	-	-

**DAY 3**				**DURATION**			**FREQUENCY**			**INTENSITY**		
**GCS**	**FOUR**	**Block**	**Time**	**Visual**	**Perm.test**	**Bayes**	**Visual**	**Perm.test**	**Bayes**	**Visual**	**Perm.test**	**Bayes**
6	8	1	18:20 PM	-	-	+	-	-	-	+	-	+
		2	20:29 PM	+	+	++	+	-	-	+	+	++++
		3	21:15 PM	+	-	-	+	-	-	+	+	++++
		4	23:14 PM	-	-	-	+	-	-	-	-	-
		5	01:05 AM	+	-	-	-	-	-	-	-	-
		6	03:13 AM	+	+	++++	+	+	+++	+	+	++++
		7	05:22 AM	+	-	++	+	-	-	+	-	-
		8	06:09 AM	-	-	-	-	-	-	+	+	+++
		9	07:56 AM	+	+	++++	+	+	++	+	+	++
		10	10:11 AM	+	+	+++	+	-	+	+	+	++++
**OUTCOME: Death (withdrawal of life support)**												

Table S3. Summary of the P3a results in Patient 2. + indicates a positive result, - a negative result. For Bayes column, + anecdotal evidence; ++ moderate evidence; +++ strong evidence and ++++ very strong to extreme evidence

**DAY 0**				**DURATION**			**FREQUENCY**			**INTENSITY**		
**GCS**	**FOUR**	**Block**	**Time**	**Visual**	**Perm. test**	**Bayes**	**Visual**	**Perm. test**	**Bayes**	**Visual**	**Perm. test**	**Bayes**
5	5	1	21:15 PM	-	-	-	-	-	-	-	-	-
		2	12:14 AM	-	-	-	+	-	-	-	-	-
		3	02:24 AM	+	-	-	+	-	-	+	+	++++
		4	4:51 AM	-	-	-	+	-	-	+	-	+
		5	07:00 AM	-	-	-	-	-	-	-	-	-
		6	08:00 AM	+	-	-	+	-	-	-	-	-
		7	10:09 AM	-	-	-	-	-	-	+	+	+++
		8	11:48 AM	-	-	-	+	-	-	-	-	-
		9	12:28 AM	+	-	-	+	-	-	-	-	-
		10	02:37 PM	-	-	-	-	-	-	-	-	-
**DAY 3**				**DURATION**			**FREQUENCY**			**INTENSITY**		
**GCS**	**FOUR**	**Block**	**Time**	**Visual**	**Perm. test**	**Bayes**	**Visual**	**Perm. test**	**Bayes**	**Visual**	**Perm. test**	**Bayes**
9	9	1	18:34 PM	+	+	+++	-	-	-	-	-	-
		2	20:48 PM	+	+	++	-	-	-	+	-	-
**OUTCOME: Good recovery**												

Table S4. Summary of the P3a results in Patient 3. + indicates a positive result, - a negative result. For Bayes column, + anecdotal evidence; ++ moderate evidence; +++ strong evidence and ++++ very strong to extreme evidence

**DAY 0**				**DURATION**			**FREQUENCY**			**INTENSITY**		
**GCS**	**FOUR**	**Block**	**Time**	**Visual**	**Perm. test**	**Bayes**	**Visual**	**Perm. test**	**Bayes**	**Visual**	**Perm. test**	**Bayes**
4	5	1	14:52 PM	+	-	+	-	-	-	+	-	++
		2	16:44 PM	-	-	-	+	-	-	+	-	-
		3	18:46 PM	-	-	-	+	-	-	-	-	-
		4	20:55 PM	-	-	-	-	-	-	+	-	-
		5	21:36 PM	+	-	-	-	-	-	+	-	-
		6	23:25 PM	+	-	-	-	-	-	-	-	-
		7	01:34 AM	-	-	-	+	-	-	+	+	++
		8	02:10 AM	-	-	-	+	+	++	+	+	++
		9	05:24 AM	-	-	-	-	-	-	-	-	-
		10	07:09 AM	-	-	-	-	-	-	-	-	-
**DAY 3**				**DURATION**			**FREQUENCY**			**INTENSITY**		
**GCS**	**FOUR**	**Block**	**Time**	**Visual**	**Perm. test**	**Bayes**	**Visual**	**Perm. test**	**Bayes**	**Visual**	**Perm. test**	**Bayes**
7	5	1	20:46 PM	-	-	-	+	-	-	+	-	++
		2	21:45 PM	+	+	++++	+	+	++++	-	-	-
		3	23:29 PM	-	-	-	+	-	-	-	-	-
		4	01:38 AM	-	-	-	-	-	-	-	-	-
		5	04:33 AM	-	-	-	-	-	-	+	-	+
		6	06:21 AM	-	-	-	-	-	-	-	-	-
**OUTCOME: UWS**												

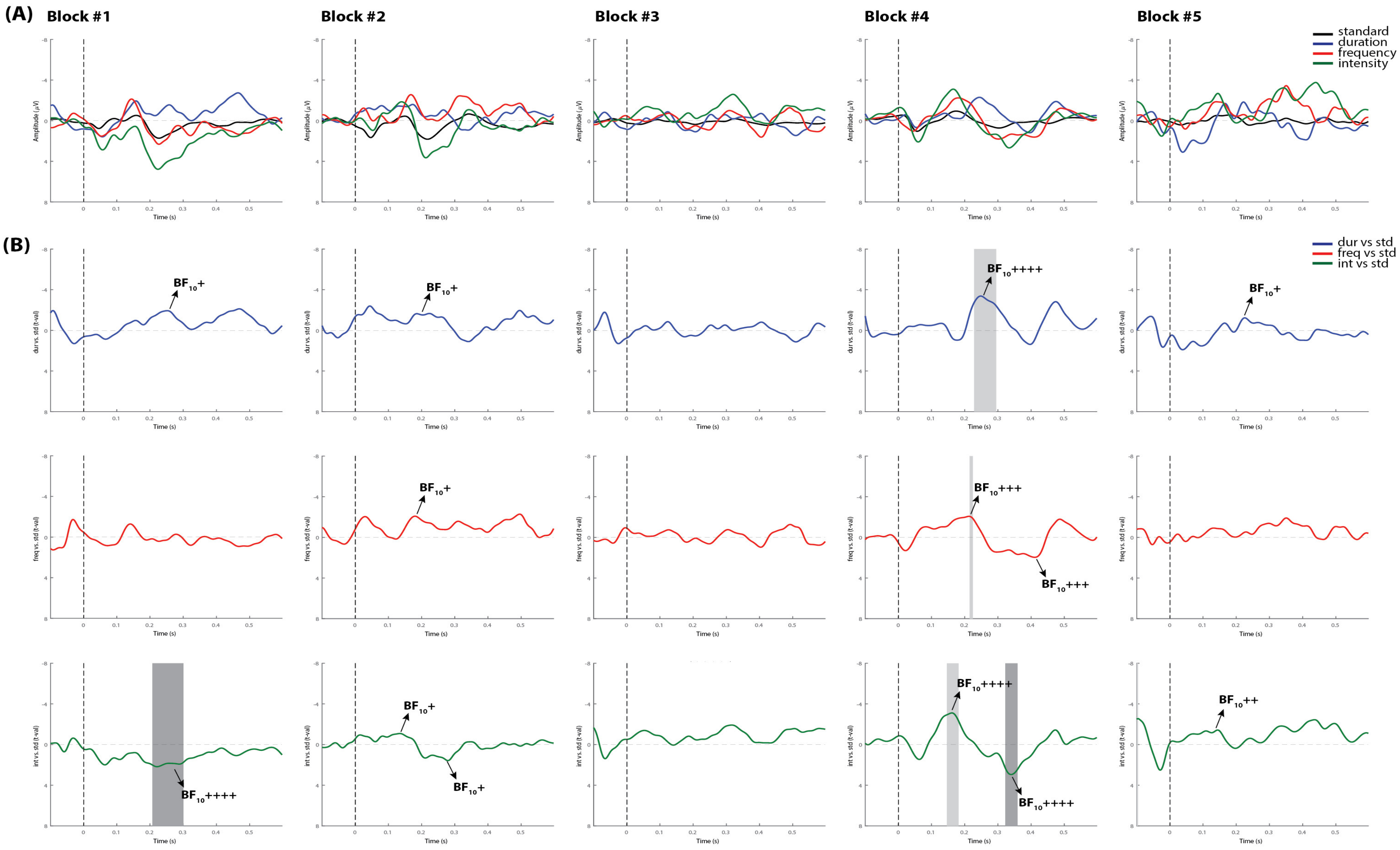

Figure S1. Individual ERPs and statistical findings of Patient 1 on day 0. (A) Individual ERPs across blocks. (B) Time course of the difference between deviants and standard stimuli expressed in units of t-values. Significant intervals for negative components are denoted by a light gray area, and those for positive components are denoted by a dark gray area. Black arrows show the latency of maximum Bayes factors and the strength of evidence for $H1$: + anecdotal; ++ moderate; +++ strong and ++++ very strong to extreme

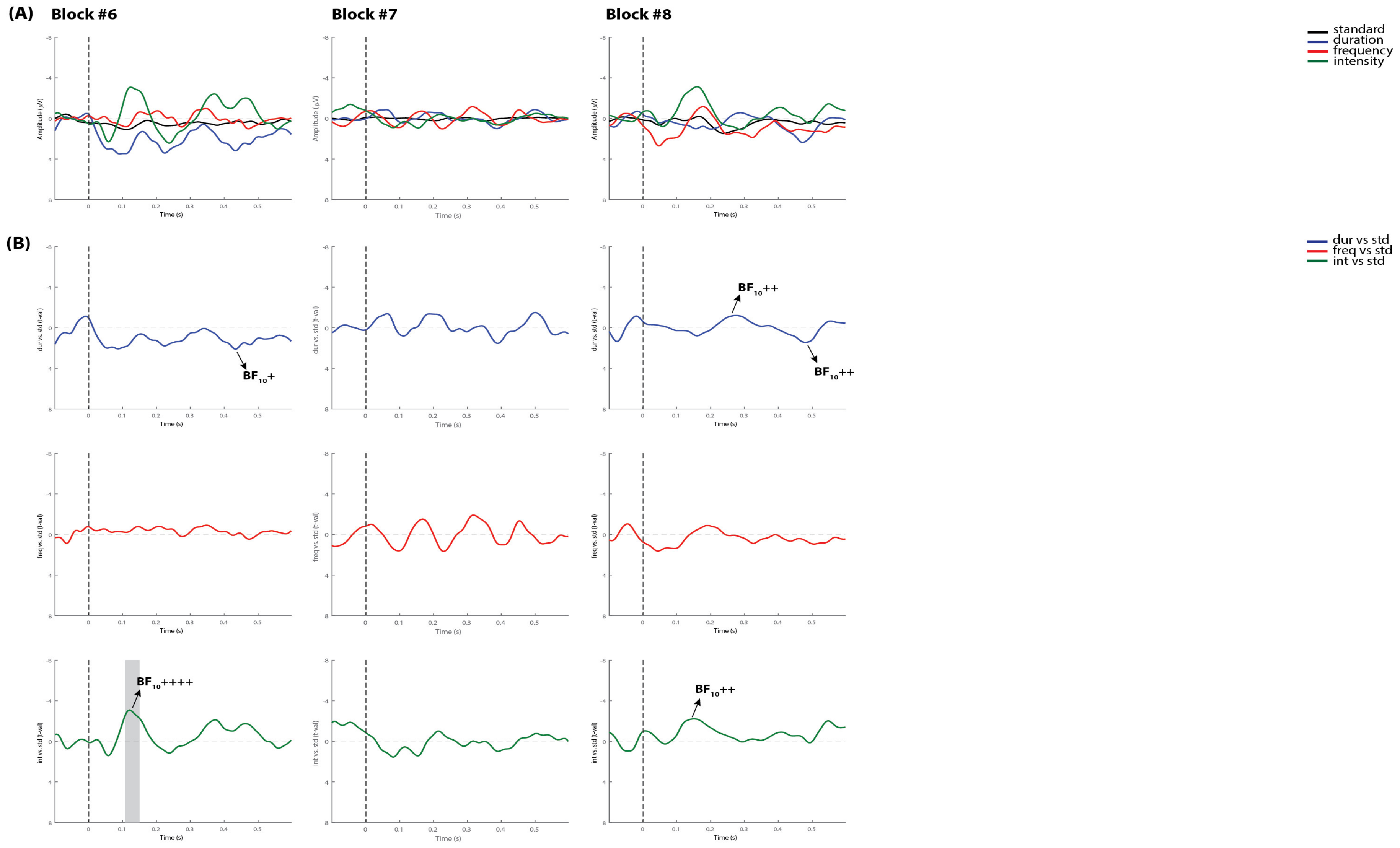

Figure S2. Individual ERPs and statistical findings of Patient 1 on day 0 (continuation). (A) Individual ERPs across blocks. (B) Time course of the difference between deviants and standard stimuli expressed in units of t-values. Significant intervals for negative components are denoted by a light gray area, and those for positive components are denoted by a dark gray area. Black arrows show the latency of maximum Bayes factors and the strength of evidence for $H1$: + anecdotal; ++ moderate; +++ strong and ++++ very strong to extreme

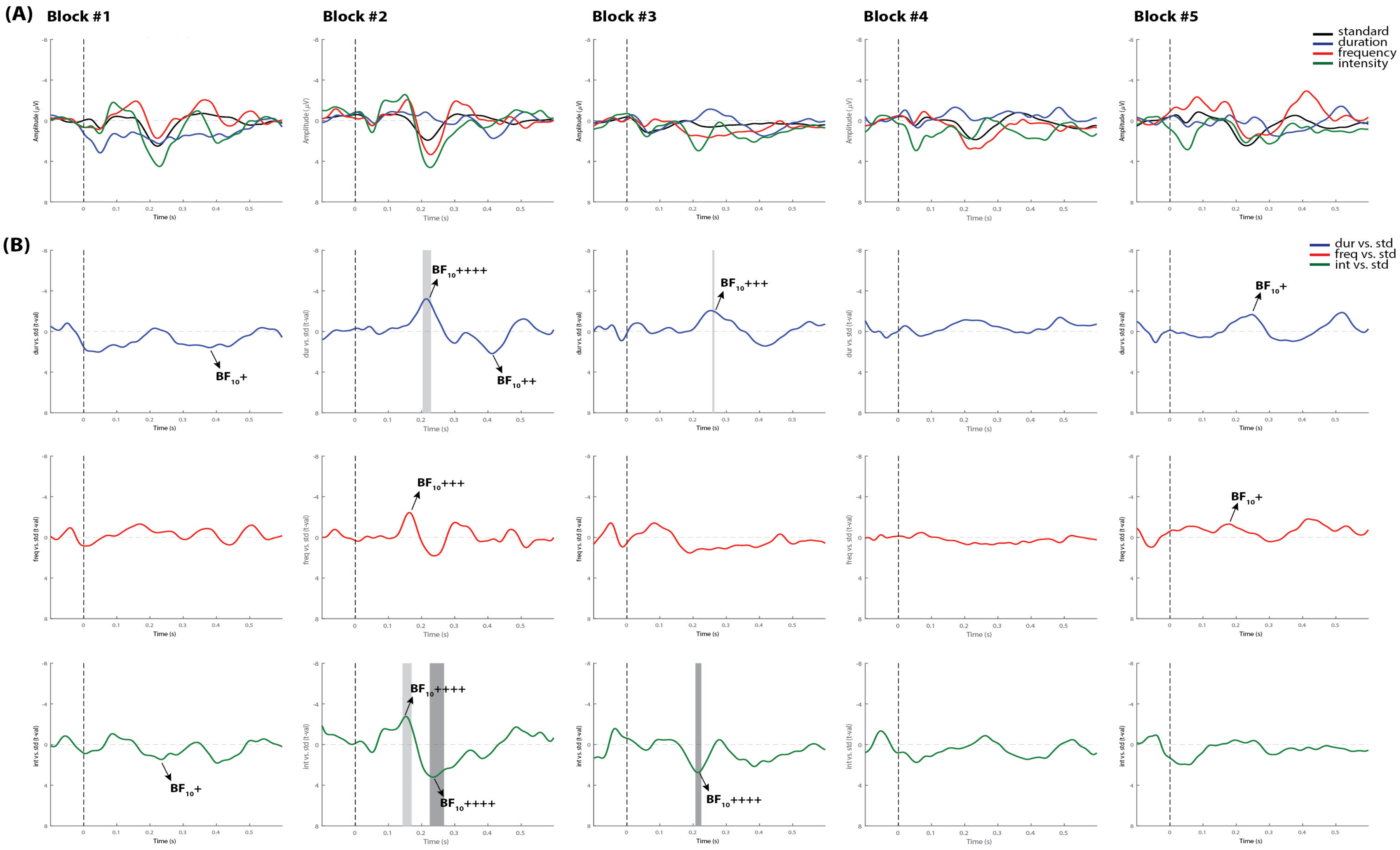

Figure S3. Individual ERPs and statistical findings of Patient 1 on day 3. (A) Individual ERPs across blocks. (B) Time course of the difference between deviants and standard stimuli expressed in units of *t*-values. Significant intervals for negative components are denoted by a light gray area, and those for positive components are denoted by a dark gray area. Black arrows show the latency of maximum Bayes factors and the strength of evidence for $H1$: + anecdotal; ++ moderate; +++ strong and ++++ very strong to extreme

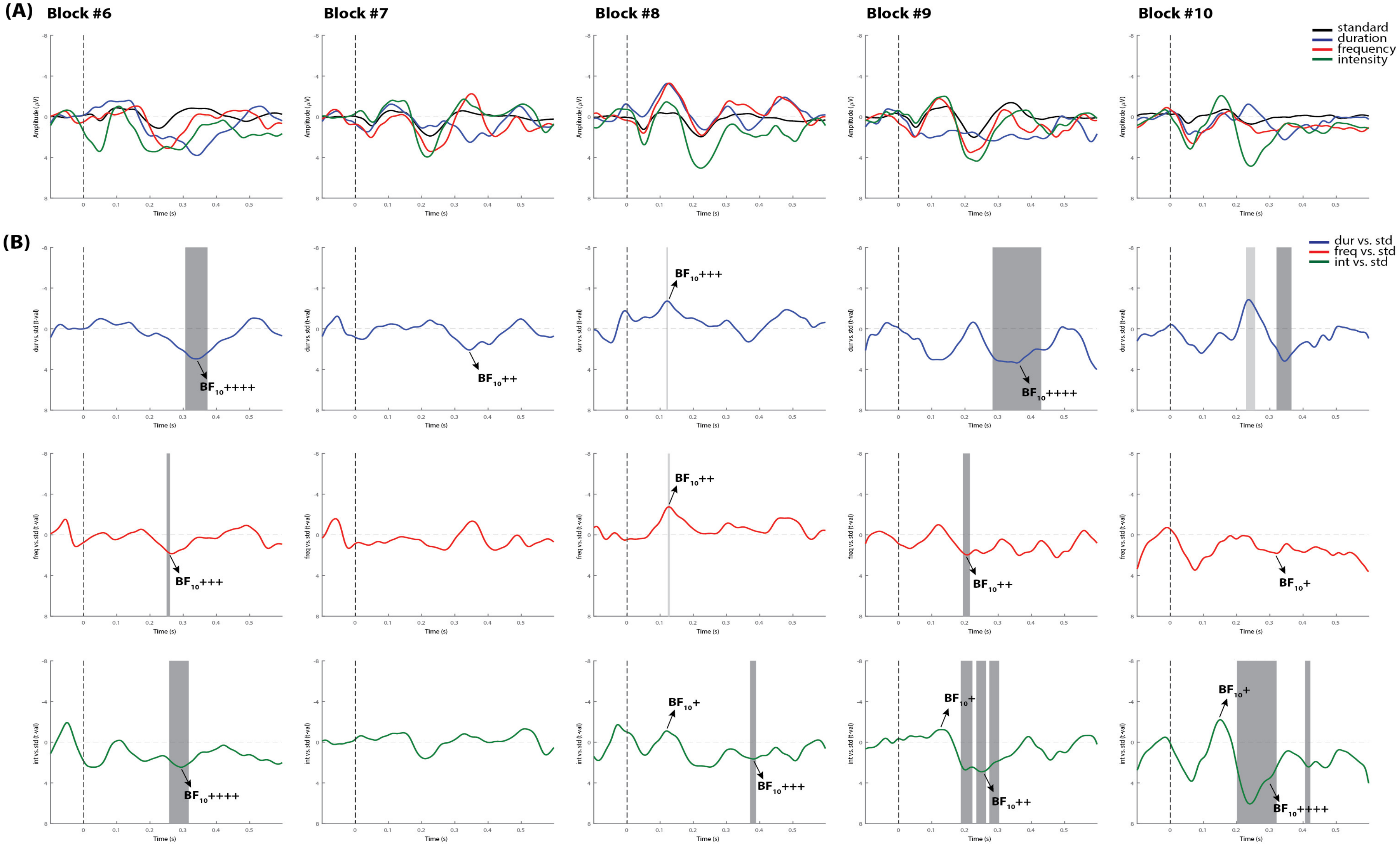

Figure S4. Individual ERPs and statistical findings of Patient 1 on day 3 (continuation). (A) Individual ERPs across blocks. (B) Time course of the difference between deviants and standard stimuli expressed in units of t-values. Significant intervals for negative components are denoted by a light gray area, and those for positive components are denoted by a dark gray area. Black arrows show the latency of maximum Bayes factors and the strength of evidence for $H1$: + anecdotal; ++ moderate; +++ strong and ++++ very strong to extreme

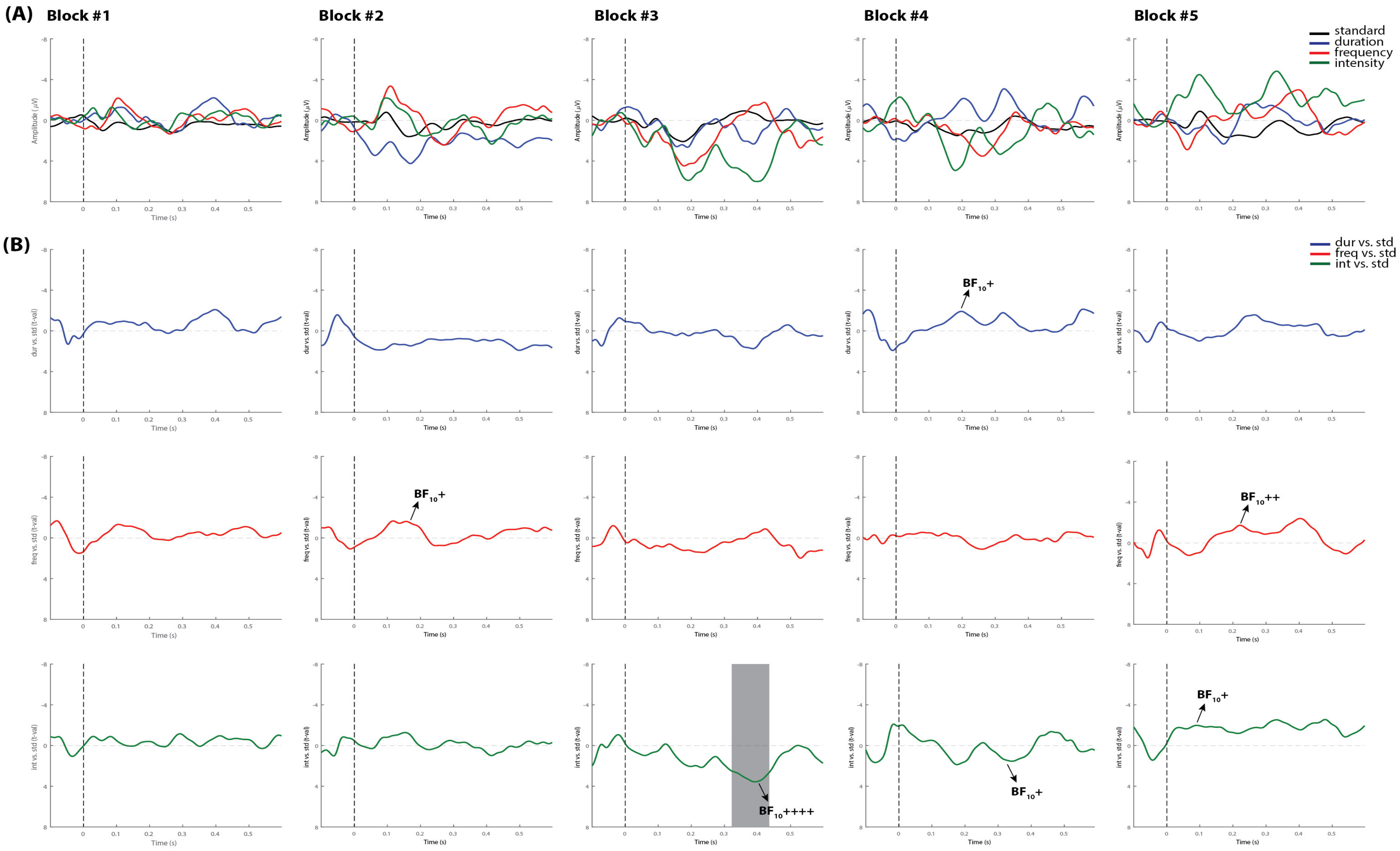

Figure S5. Individual ERPs and statistical findings of Patient 2 on day 0. (A) Individual ERPs across blocks. (B) Time course of the difference between deviants and standard stimuli expressed in units of *t*-values. Significant intervals for negative components are denoted by a light gray area, and those for positive components are denoted by a dark gray area. Black arrows show the latency of maximum Bayes factors and the strength of evidence for $H1$: + anecdotal; ++ moderate; +++ strong and ++++ very strong to extreme

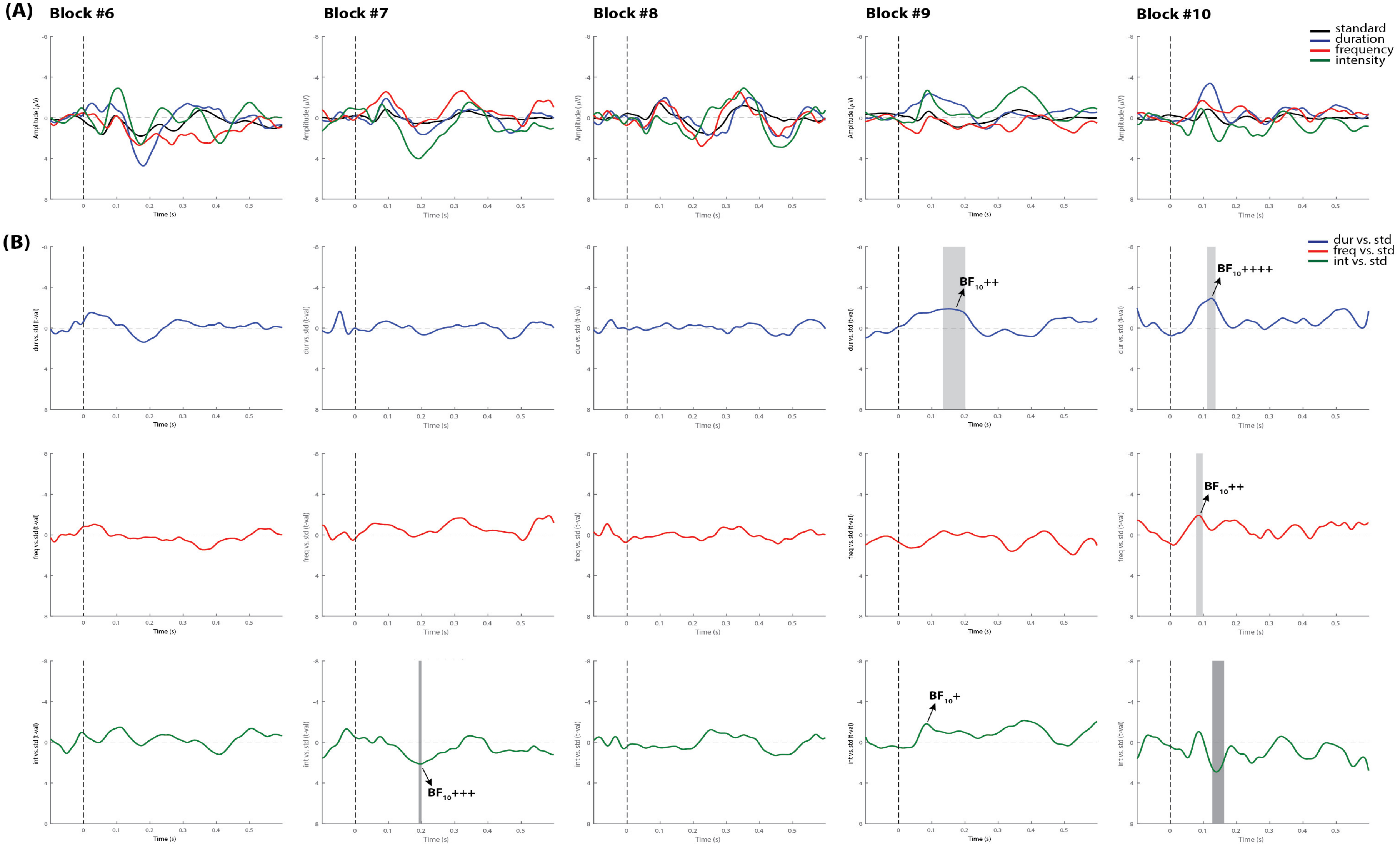

Figure S6. Individual ERPs and statistical findings of Patient 2 on day 0 (continuation). (A) Individual ERPs across blocks. (B) Time course of the difference between deviants and standard stimuli expressed in units of t-values. Significant intervals for negative components are denoted by a light gray area, and those for positive components are denoted by a dark gray area. Black arrows show the latency of maximum Bayes factors and the strength of evidence for $H1$: + anecdotal; ++ moderate; +++ strong and ++++ very strong to extreme

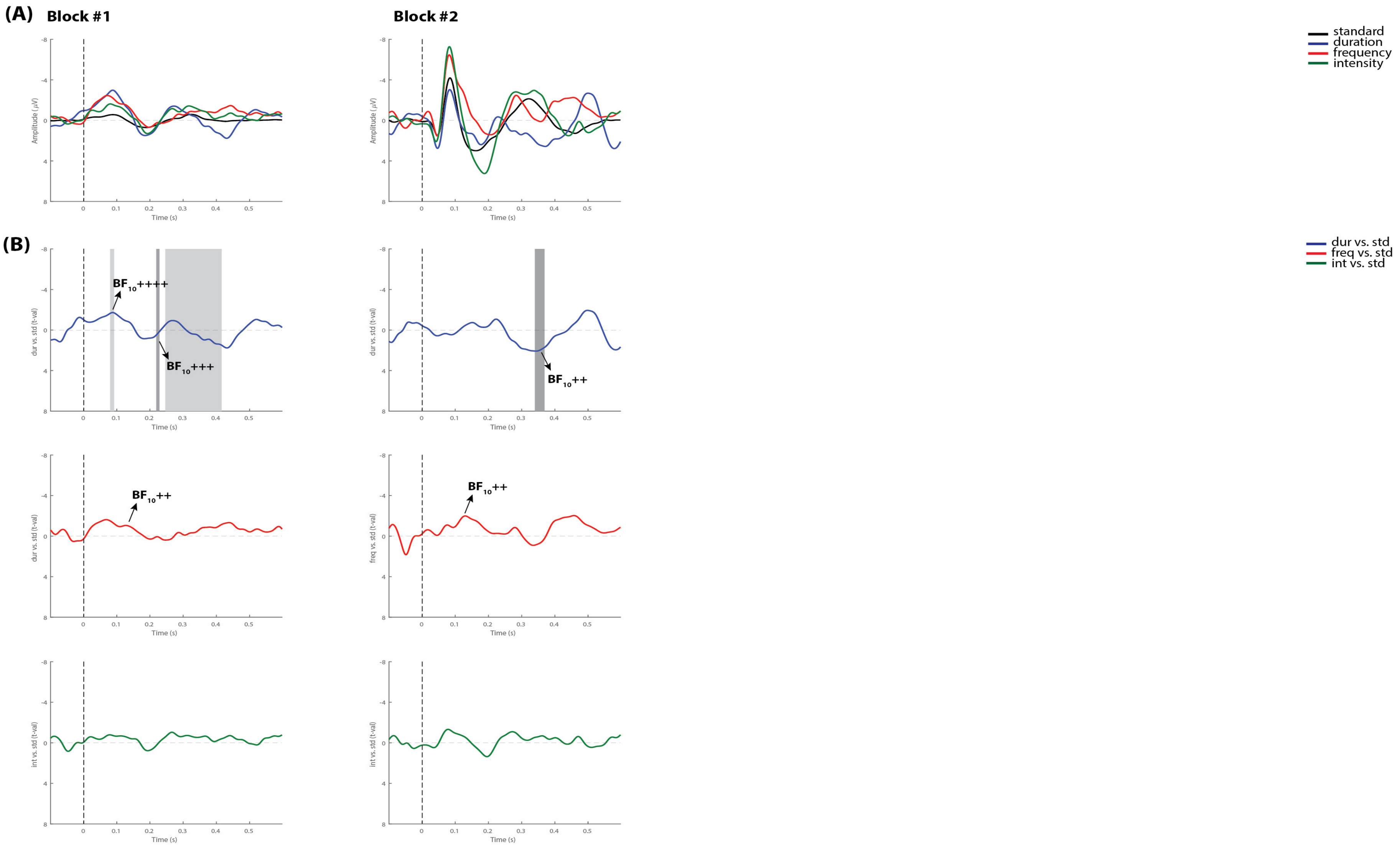

Figure S7. Individual ERPs and statistical findings of Patient 2 on day 3. (A) Individual ERPs across blocks. (B) Time course of the difference between deviants and standard stimuli expressed in units of t-values. Significant intervals for negative components are denoted by a light gray area, and those for positive components are denoted by a dark gray area. Black arrows show the latency of maximum Bayes factors and the strength of evidence for $H1$: + anecdotal; ++ moderate; +++ strong and ++++ very strong to extreme

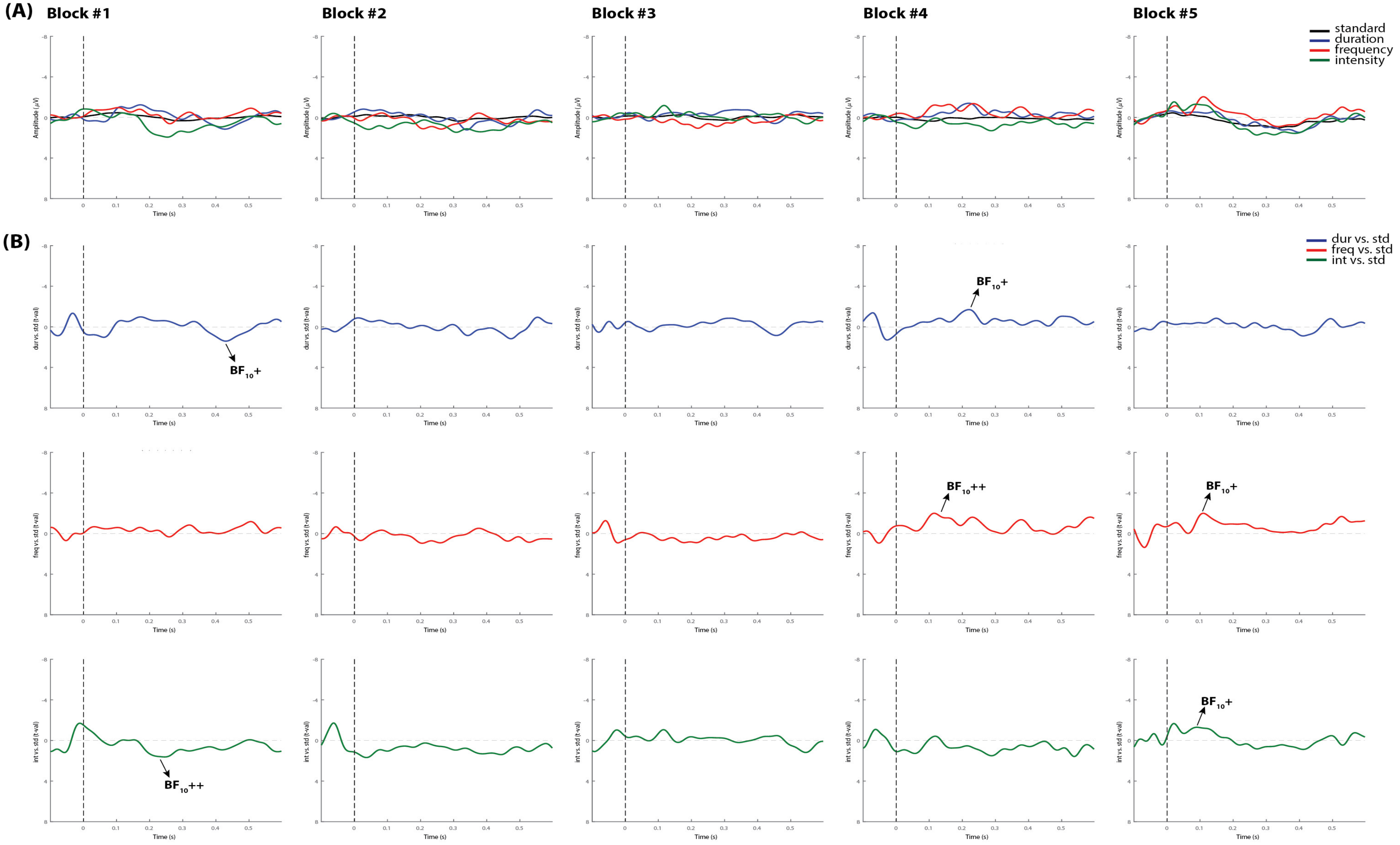

Figure S8. Individual ERPs and statistical findings of Patient 3 on day 0. (A) Individual ERPs across blocks. (B) Time course of the difference between deviants and standard stimuli expressed in units of *t*-values. Significant intervals for negative components are denoted by a light gray area, and those for positive components are denoted by a dark gray area. Black arrows show the latency of maximum Bayes factors and the strength of evidence for $H1$: + anecdotal; ++ moderate; +++ strong and ++++ very strong to extreme

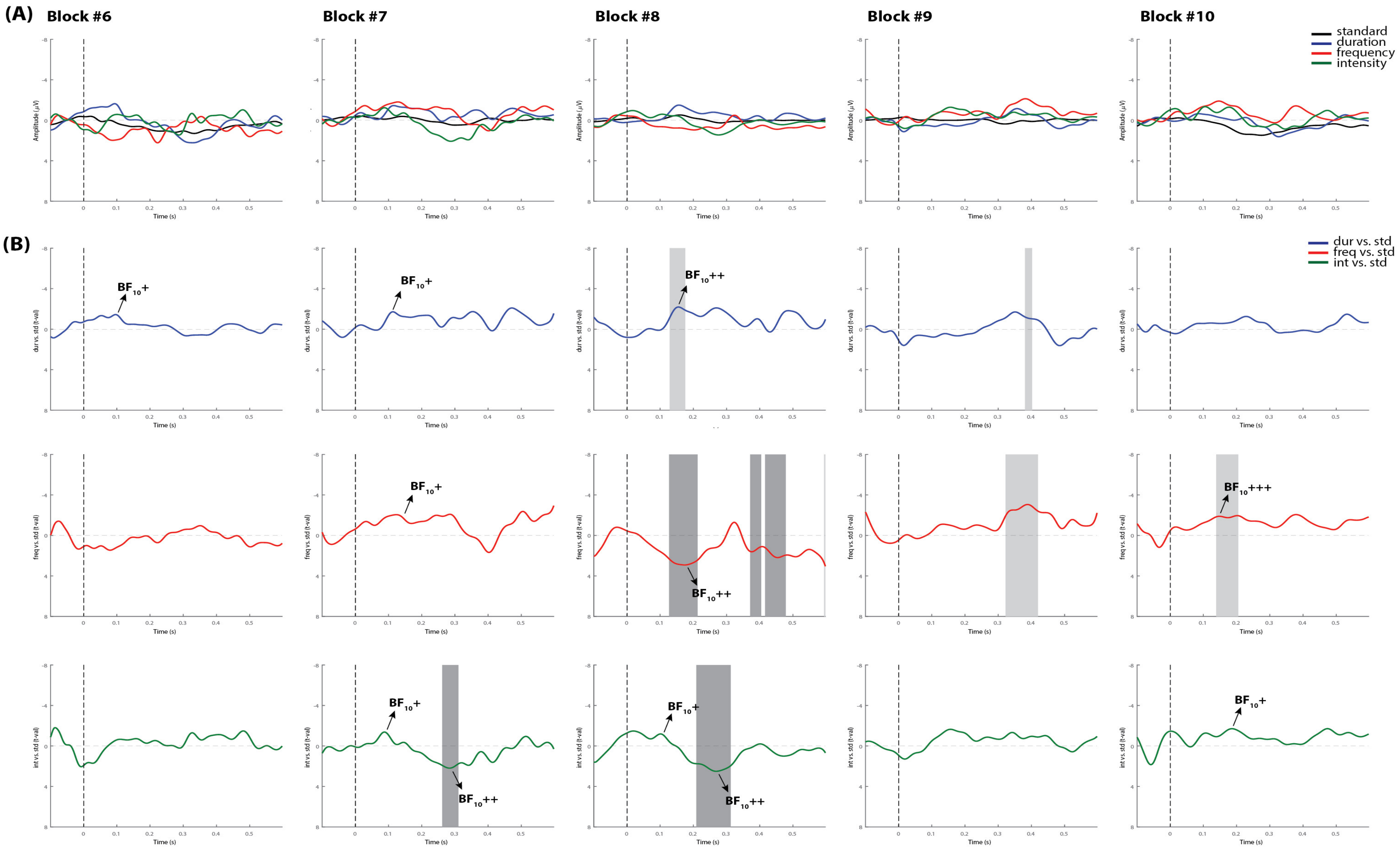

Figure S9. Individual ERPs and statistical findings of Patient 3 on day 0 (continuation). (A) Individual ERPs across blocks. (B) Time course of the difference between deviants and standard stimuli expressed in units of t-values. Significant intervals for negative components are denoted by a light gray area, and those for positive components are denoted by a dark gray area. Black arrows show the latency of maximum Bayes factors and the strength of evidence for $H1$: + anecdotal; ++ moderate; +++ strong and ++++ very strong to extreme

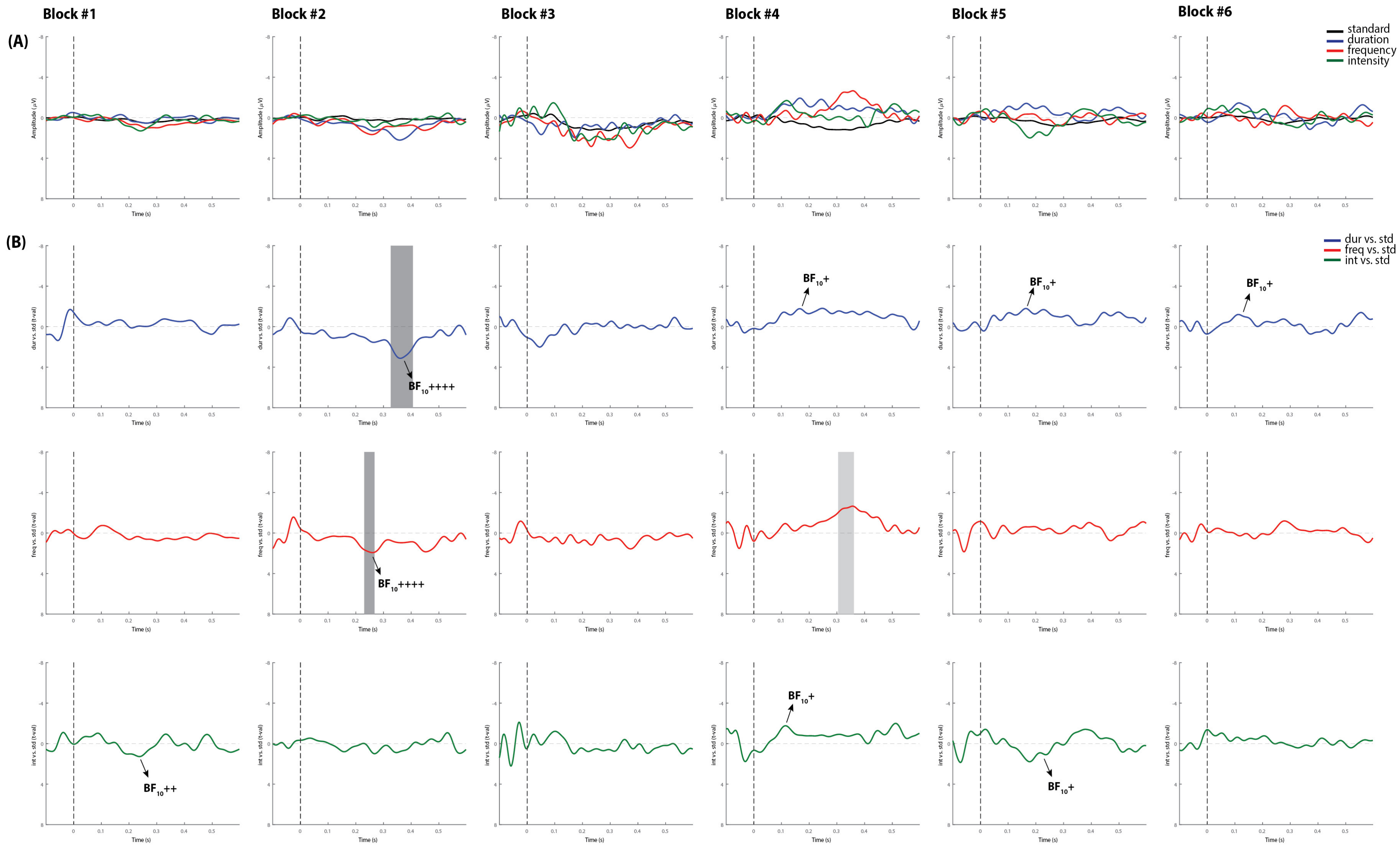

Figure S10. Individual ERPs and statistical findings of Patient 3 on day 3. (A) Individual ERPs across blocks. (B) Time course of the difference between deviants and standard stimuli expressed in units of t-values. Significant intervals for negative components are denoted by a light gray area, and those for positive components are denoted by a dark gray area. Black arrows show the latency of maximum Bayes factors and the strength of evidence for $H1$: + anecdotal; ++ moderate; +++ strong and ++++ very strong to extreme
